# COPD Diagnosis: Time for Disruption

**DOI:** 10.3390/jcm10204660

**Published:** 2021-10-11

**Authors:** Emiel F. M. Wouters, Marie K. Breyer, Robab Breyer-Kohansal, Sylvia Hartl

**Affiliations:** 1 Ludwig Boltzmann Institute for Lung Health, 1140 Vienna, Austria; Marie-Kathrin.breyer@lunghealth.lbg.ac.at (M.K.B.); Robab.Breyer-Kohansal@lunghealth.lbg.ac.at (R.B.-K.); hartlsylvia@icloud.com (S.H.); 2 Department of Respiratory Medicine, Maastricht University Medical Center, 6229 HX Maastricht, The Netherlands

**Keywords:** COPD, taxonomy, early disease, airflow limitation

## Abstract

Articulating a satisfactory definition of a disease is surprisingly difficult. Despite the alarming individual, societal and economic burden of chronic obstructive pulmonary disease (COPD), diagnosis is still largely based on a physiologically dominated disease conception, with spirometrically determined airflow limitation as a cardinal feature of the disease. The diagnostic inaccuracy and insensitivity of this physiological disease definition is reviewed considering scientific developments of imaging of the respiratory system in particular. Disease must be approached as a fluid concept in response to new scientific and medical discoveries, but labelling as well as mislabelling someone as diseased, will have enormous individual, social and financial implications. Nosology of COPD urgently needs to dynamically integrate more sensitive diagnostic procedures to detect the breadth of abnormalities early in the disease process. Integration of broader information for the identification of abnormalities in the respiratory system is a cornerstone for research models of underlying pathomechanisms to create a breakthrough in research.

## 1. Introduction

Chronic obstructive pulmonary disease (COPD) is a public health challenge associated with a significant morbidity and mortality. COPD led to 3.2 million deaths in 2015 and was estimated to become the third most common cause of death in 2030 [1,2,3]. Worldwide prevalence is estimated around 10% and 174.5 million people are diagnosed with COPD [1,4]. This represents an increase in prevalence of 44.2% and an increase in mortality of 11.6% since 1990 [1]. COPD is estimated to be the 9th most influential disease in increasing disability-adjusted life years [5].

Besides the disease burden, COPD is associated with substantial economic costs. In the European Union, the total direct costs are estimated to be about 3% (EUR 38.6 billion) of the total health care budget [6]. Similar figures are reported for COPD in the United States: in 2010, it caused USD 30 billion in direct medical costs and USD 20 billion in indirect costs [7]. The actual societal costs are substantially higher due to excess health care utilisation, work absence and premature retirement [8].

Confronted with all these alarming figures and predictions, the definition of COPD is still largely descriptive and in the absence of a clear etiological origin dominated by non-remitting airflow obstruction as the pathophysiological characteristic in which the diseased group differs from the norm [9,10,11]. Otherwise, considering a disease as a description of those abnormal phenomena observed in a group of organisms with a disturbed function or structure, four principal methods are described to define the disease condition: clinical, morbid anatomical, functional or physiological and aetiological. Defining the disease as a physiological malfunction independent of subjective experience and social conventions fits with the nosological approach in the mid-20th century [9,12,13]. At the turn of the last century, the Global initiative for Chronic Obstructive Lung Disease (GOLD) confirmed the definition of COPD as a disease state characterised by airflow limitation which is not fully reversible. This airflow limitation is usually progressive and associated with an abnormal inflammatory response of the lungs to noxious particles or gases [14]. From the beginning, patients with symptoms of chronic bronchitis or emphysema who do not have airflow obstruction are excluded and removed from the diagnosis of COPD [10,15]. In the latest definition, illness characteristics as well as alveolar abnormalities are included in the definition of COPD, but GOLD sticks to the criterion of airflow limitation with a cut-off value of 0.70 for the ratio of forced expiratory volume/forced vital capacity, independent from the individual respiratory journey of the patient [3,12]. 

Disease must be considered as a fluid concept influenced by societal and cultural attitudes that change with time and in response to new scientific and medical discoveries [16]. One of these developments is, besides the implementation of new physiological measures such as forced oscillations and multiple-breath nitrogen washout, the enormous amount of information provided by non-invasive imaging of the respiratory system. The aim of this perspective is to review the current evidence of the historical physiological condition of airflow obstruction and to summarise the information provided by a variety of imaging modalities. In particular, chest computed tomography (CT) provides insight into structural as well as pathophysiological pulmonary parameters.

## 2. The Airflow Tunnel View

The invention of the spirometer and the timed measurement of forced exhalation of expired air has revolutionised the diagnostic approach of COPD in the 19th century [17,18,19,20]. The presence of an obstructive ventilatory defect was considered as a hallmark of COPD [21]. An obstructive ventilatory defect was defined as a disproportionate reduction in maximal airflow from the lung in relation to the maximal volume that can be displaced from the lung [22,23,24]. The earliest change associated with airflow obstruction is thought to be a slowing in the terminal portion of the spirogram even when the initial part of the spirogram is barely affected. As the airway disease becomes more advanced, timed segments of the spirogram such as the FEV_1_ will be reduced out of proportion to the reduction in VC [25]. Ideally, the principles of clinical decision making should be applied in case of pulmonary function interpretation: the post-test probability of disease will be estimated after taking into consideration the pre-test probability of disease, the quality of the test results, the downside of false-positive and false-negative interpretation and the comparison of the test results themselves with reference values [25,26]. The most important parameter in identifying an obstructive impairment is the forced expiratory volume in one second (FEV_1_)/vital capacity (VC) ratio. The presence of an obstructive ventilatory effect is defined by a reduced FEV_1_/VC ratio below the 5th percentile of the predicted value [25].

However, to increase the awareness of diagnosing COPD, and driven by a desire for simplicity, GOLD has pragmatically used a fixed FEV_1_/VC ratio of 0.70 as the threshold for defining COPD to help both the diagnosis and epidemiological study of COPD [14]. In addition, on the basis of post-bronchodilator FEV_1_ values, GOLD classified COPD into four categories (stage 1 to 4) [14]. This fixed ratio, although easy to remember, created an area of controversy from the beginning as it does not take into account the age-related decline in the FEV_1_/VC ratio, possibly leading to an under-diagnosis of COPD in younger adults and an over-diagnosis of COPD in elderly subjects [27]. Later on, the American Thoracic Society (ATS)/European Respiratory Society (ERS) guidelines recommended that the lower limit of normal (LLN) should be used to classify obstruction based on spirometry [25]. While previous longitudinal data reported that LLN may miss subjects at risk, a recent analysis of the data from a population-based Canadian cohort demonstrated that airflow limitation defined solely by a fixed ratio or LNN is only weakly and variably associated with patient-reported outcomes such as symptoms, disability, impaired health status and exacerbations. Patients meeting both criteria had more consistent and greater increases in risk for COPD-related outcomes while patients meeting either criterion, and in addition, have a low FEV_1_, were the most likely to experience poor outcomes [28,29]. Using pooled data from multiple NIH cohorts, Bhatt et al. confirmed that a fixed FEV_1_/FVC ratio < 0.70 provides discrimination of COPD-related hospitalisation and mortality that was not significantly different or was not more accurate than other fixed thresholds and the LNN. The authors conclude that these results support the use of the FEV_1_/FVC ratio < 0.70 to identify individuals at risk of clinically significant COPD [30]. Others reported that FEV_1_ itself is one of the most powerful predictors of clinically relevant outcomes including symptoms, exacerbations and mortality [31,32]. Although these and other data stress the importance of airflow limitation as a marker of disease burden, no information is provided about the sensitivity to detect early disease-related abnormalities as a gold standard is lacking [33,34,35]. This bias of an imperfect gold standard must be borne in mind when interpreting airflow limitation as the only dominant marker of COPD diagnosis [36].

The need for a gold standard is also stressed by new insights on the range of lung-function trajectories throughout life. In asymptomatic, non-smoking males, longitudinal lung function data demonstrated that after a prolonged plateau phase from ages 23–35, decline in lung function began in two phases that averaged about −20 and −30 mL/year [37]. In contrast to the findings of Fletcher and Peto, Tager et al. reported more than thirty years ago that the rate of lung function decline in smokers is only slightly greater than that observed for non-smokers, but this decline begins in the early part of the third decade while this group does not have a plateau phase. These data already suggested that the major effect of smoking on lung function decline involves the premature onset of a normal decline in function, irrespective of gender [37,38]. Furthermore, recent data confirm that accelerated decline in FEV_1_ is not an obligate feature to reach the spirometric criterion of COPD [39]. Lange et al. reported that lung-function value reached in early adulthood is important with respect to the diagnosis of COPD later in life; half of the persons presenting with airflow limitation followed the paradigm which implied a rapid decline in FEV_1_ from a normal level of lung function in early adulthood, whereas the other half had a rather normal decline in FEV_1_ but started from a low initial value of FEV_1_. When rapid decline in FEV_1_ is generally considered as a characteristic of disease activity in COPD, at least it must be assumed that this is not the case in half of the COPD population analyses in the three independent cohorts studied by Lange et al. questioning the specificity of the FEV_1_/VC ratio < 0.70 as a criterion for the “disease” condition of COPD [38]. These alternative courses of lung function changes were already suggested in previous literature [40]. Others confirmed that low peak lung function in early adulthood is common in the general population and that trans-generational effects contribute to this early adulthood condition [41]. The concept of multiple lung function trajectories in smokers with and without COPD is supported by modelling longitudinal data of the Normative Age Study and from the COPD Gene Study [42].

Interestingly, a recent meta-analysis reported that even late adolescents and young adults born very preterm (mean gestational age: 28.3 weeks) or with extremely low birth weight (mean birth weight: 1054 g) had substantially reduced airflow. These data illustrate that these infants are not achieving their full airway growth potential and that even if the maximal expiratory flow declines at a normal rate with age in adulthood, proportionally more will achieve the fixed cut-off ratio of 0.70 [41,42,43]. The emerging role of intra- and extra-uterine environmental insults as predisposing factors to develop airflow limitation later in life is now well appreciated. Changes in gene expression not only impair harmonious lung development but also predispose to airspace enlargement and emphysema [44,45].

Besides specificity, sensitivity to detect and address early symptoms in persons at risk for the disease condition is an important characteristic of a marker of disease. Previous studies already reported data concerning exacerbation-like events in individuals without airflow limitation. Tan et al. reported that the occurrence of these events is half of the frequency in non-COPD individuals compared with those with COPD. In the non-COPD group, these events were independently associated with female gender, presence of wheezing, attenuated self-perceived health, and remarkably, the use of respiratory medications: 24.2% of these non-COPD patients were using respiratory medications [46]. In a follow-up study of 8246 participants of the COPD Gene cohort, Bowler et al. reported that acute episodes of respiratory disease are common in current and former smokers without COPD, particularly when there is a history of prior events and poor respiratory health status [47]. A second study from this COPD Gene cohort, demonstrated that respiratory symptoms are highly common in smokers without spirometric COPD and that these subjects had manifested clinical disease with dyspnoea, chronic bronchitis, lower walk distance and worse quality of life [48]. Woodruff et al. confirmed these reported findings: respiratory symptoms and exacerbations are common in current and former smokers with preserved lung function; many of these persons were already being treated with respiratory medications despite a lack of evidence. Among symptomatic smokers, 42% used bronchodilators and 23% used inhaled glucocorticoids [49]. Indeed, it is important to decipher the underlying mechanisms of these respiratory symptoms [50]. In the original publication of the CIBA symposium, held in 1959, chronic bronchitis was already identified as a separate entity only based on the clinical diagnostic criterion of chronic or current cough with expectoration, not attributable to other conditions [51]. The concept of chronic cough as clinical entity with unique epidemiology in adults has led to the concept of the cough hypersensitivity syndrome [52]. At least, these studies illustrate the complexity to label the broad spectrum of respiratory symptoms and the limitations of a one size fits all approach in the diagnostic work-up.

Airflow limitation is not the only trait in COPD: in a more recent definition, both airways diseases as emphysema are considered part of the structural abnormalities in the pathophysiology of COPD [53]. Levine et al. already reported that abnormalities of ventilation distribution and gas exchange occur in mild bronchitis and asymptomatic asthma patients before other abnormalities of lung function become apparent [54]. Diffusing capacity of the lung for carbon monoxide (DLCO) as a non-invasive test of pulmonary gas transfer, and has been considered for a long time as a surrogate marker for loss of alveolar tissue [55]. In cross-sectional studies, smoking has been found to be associated with impaired DLCO [56,57]. Changes in DLCO/alveolar volume (VA) associated with stop smoking are considerably larger than can be explained by carbon monoxide back pressure, indicating that mechanisms other than irreversible increase in the size of terminal air spaces underlie these lower values in smokers [57]. From an urban population-based study, two subsets of current smokers with normal spirometry were followed over 3 years: one subset with normal diffusing capacity and one subset with low DLCO. In the normal spirometry/normal diffusing capacity group (*n* = 59), 3% developed COPD, while in the low DLCO group (*n* = 46), 22% developed COPD, suggesting that the latter group is at a more significant risk of developing COPD [58]. Only a few studies have explored longitudinal changes in pulmonary diffusing capacity in relation to smoking. In a small cohort followed over 22 years, continuing smokers resulted in a 10% fall in DLCO [59]. Two longitudinal studies over 8 years—including 543 subjects and 928 subjects, respectively— found that the decline in DLCO during follow-up increased with age, while no relationship to smoking was noticed: no difference was found in the mean slopes over time between current smokers and never smokers [60,61]. In a Norwegian community sample of 1152 subjects, followed over 9 years, a more rapid decline in DLCO was related to higher age, baseline current smoking, more pack years, larger weight and lower FEV_1_. These reported findings suggest that a direct assessment at the level of parenchymal surrogates is probably more appropriate for the detection of lung parenchymal abnormalities [62]. Furthermore, it has to be acknowledged that the heterogeneous respiratory impairment in COPD needs a larger armentarium of “lung function tests” [63].

An important limitation of the current spirometric definition of COPD is that no information can be obtained about ongoing processes in the silent or quiet zone of the lungs, the airways < 2 mm in diameter where disease can accumulate over many years without being noticed [64]. Indeed, at the time Fletcher and Peto were conducting their prospective study on the natural history of chronic bronchitis and emphysema, it was thought that the small conducting airways that connect the bronchi to the gas exchanging surface were the major site of resistance to airflow in the lower respiratory tract of normal individuals. This was based on the aerodynamic calculations on casts from human lungs conducted by Rohrer [65]. This classical concept was challenged in 1963 by the publication of Weibel on the morphometry of the human lung: he provided quantitative information that the total cross-sectional area of the conducting airways increased exponentially as the gas exchange area was approached [66]. Macklem and Mead provided the first experimental data to support this concept: they developed a method to directly measure the peripheral airway resistance by positioning a catheter in the airways < 2 mm in diameter. They showed that under normal conditions, the airways < 2 mm in diameter accounted for <10% of the total resistance to flow below the larynx [67]. However, the same airways offering so little resistance in normal lungs became the major site of obstruction to airflow in postmortem lungs affected by emphysema with narrowing and distortion of the lumens of the smaller bronchi and bronchioles and with presence of a mixture of chronic inflammation and fibrosis in the airway walls [68]. Yanai and co-workers reported data of direct central and peripheral airway resistance measurement in awake humans. The found that peripheral resistance significantly increases in patients with bronchial asthma with airflow obstruction and patients with chronic bronchitis and emphysema, confirming that peripheral airways are the predominant site of airflow obstruction [69].

The concept of small airways disease is now widely accepted but developments were hampered by lack of adequate methodology to detect these disease abnormalities in an early phase [70]. Measurement of lung mechanics with forced oscillation techniques now has the potential to detect these peripheral resistance changes with greater sensitivity than spirometric measurement [71].

At least, current evidence illustrates that strict application of spirometry as the only physiological measure and the strict definition of obstruction based on FEV_1_/FVC ratio does not allow an adequate classification, even in patients with significant morbidity. It does not allow the detection or diagnosis of emphysema as a morbid anatomical change as a characteristic component of COPD. It does not allow accurate diagnosis of the systemic manifestations of the COPD syndrome, neither identification of COPD endotypes. Sticking on the current conventional diagnostic labelling of COPD by spirometry only reflects a tunnel view in the diagnostic work-up of patients manifesting a broad spectrum of respiratory complaints.

## 3. Imaging of the Lungs: A New Perspective to Redefine Copd

Chest computed tomography (CT) is a widely available non-invasive imaging modality that provides insight into structural and pathophysiological pulmonary parameters [72]. Many persons at risk and with COPD already undergo a chest CT for lung cancer screening or to evaluate pulmonary nodules detected on a chest X-ray. However, the wealth of chest CT scan data is not consistently used in clinical practice and incorporated into clinical diagnosis and management of COPD [73]. Emphysema can be identified based on measuring lung density in Hounsfield units as well as airway wall thickness and airway counts.

More than 25 years ago, Remy-Jardin already described morphological effects of cigarette smoking on airways and lung parenchyma in healthy adult volunteers. They reported parenchymal abnormalities in healthy smokers, varying from parenchymal micro-noduli to emphysema and areas of ground-glass attenuation [74]. In a large group of male smokers without (61.7%) or with mild COPD from a Lung Cancer Screening trial, CT imaging grouped the majority of subjects either into a dominant emphysematous group, a group with air trapping or with CT-defined airway wall thickening. Airway wall thickening dominance was associated with younger age, higher body mass index, more wheezing and lower FEV_1_% predicted. The emphysema subjects had lower FEV_1_/FVC ratio and more impaired gas transfer [75]. In recent years, large observational studies such as COPD Gene and CanCOLD significantly contributed to a better understanding of respiratory-related impairments in smokers with normal spirometry [76]. In so-called GOLD 0 participants, more than 40% had CT evidence of emphysema or airway thickening [48]. These data were confirmed in the Canadian cohort: respiratory bronchiolitis as well as air trapping was more prevalent in ever-smokers with normal lung function than in mild or moderate COPD patients. Bronchial wall thickening was found in more than half of smokers without COPD. Similarly, the proportion of individuals with emphysema was elevated in ever-smokers with normal lung function (30%) versus the normal population (11%). Intriguingly, this presence of emphysema on CT was associated with chronic cough and phlegm production, wheeze, dyspnoea, impaired health status and even increased risk of more than two exacerbations over 12 months [77]. In the cohort of symptomatic current or former smokers with preserved pulmonary function, Woodruff et al. also reported a greater airway wall thickening in these individuals but no predominance of emphysematous features [49]. All these data clearly illustrate that CT has the potential of identifying structural parenchymal and airway abnormalities even in smokers with preserved lung function. 

Parametric response mapping (PRM) was introduced some years ago as a quantitative imaging biomarker to assess the phenotypic contributions of functional small airway disease (fSAD) and emphysema in COPD when applied to inspiratory and expiratory CT images. Based on this application, cyclic physiological respiratory states are being captured through imaging and computed on a voxel-by-voxel basis by image co-registration [78]. Using PRM fSAD is found to be present in 12.4% of smokers or former smokers with preserved lung function and increased to 39.2% in four COPD GOLD patients. Emphysema is present in a negligible portion in participants with preserved lung function. Intriguingly, high levels of CT-defined small airway abnormality in individuals without airflow limitation was associated with more rapid declines in FEV_1_ as well as in mild-to-moderate COPD stages. Contributions of small airways disease and emphysema on decline in FEV_1_ are more balanced in advanced stages of COPD [79]. Interestingly, analysis of ever-smokers without obstruction and with GOLD 1–2 COPD showed that PRM fSAD correlates with low diffusing capacity suggesting that it may detect airways transitioning to early emphysema, resulting in impaired gas exchange [80]. Based on CT scanning, two distinct trajectories of disease progression could be identified: the tissue → airway subtype and an airway → tissue subtype. In the first subtype, small airway dysfunction and emphysema precede large airway wall abnormalities while in the other subtype large airway wall abnormalities precede emphysema and small airway dysfunction [81]. Intriguingly, dysanapsis quantified on CT by calculation of the airway-to-lung ratio accounted for a greater proportion of variation in FEV_1_/FVC ratio than smoking and other COPD risk factors, suggesting the dysanapsis is even a risk factor for COPD among older adults. Furthermore, those persons with dysanapsis do not have accelerated decline in lung function either before or after the development of COPD [82].

By use of micro-CT on fixed and dried lung samples, Mc Donough et al. reported in 2011 an important reduction (72%) in terminal bronchioles in patients with severe COPD. The number of terminal bronchioles was reduced even in tissue samples that had no detectable emphysema, suggesting that terminal bronchiole obliteration precedes emphysematous tissue destruction in COPD [83]. These data were confirmed and extended by the publication of Koo et al., demonstrating that the smallest airways, the conducting terminal bronchioles and respiratory transitional bronchioles are significantly lost in the lungs of patients with mild and moderate COPD compared with age-matched smokers with normal lung function, although the lung tissue does not show emphysematous destruction. The remaining small airways have thickened walls [84]. In another paper, total airway count (TAC) was measured on in vivo CT scans in a population-based sample of participants at risk and with mild COPD. The authors reported a significant reduction in 17% in total airway count in mild COPD patients (GOLD 1) compared with at-risk participants independent of emphysema. Furthermore, among all CT measurements investigated, TAC had the greatest effect on pulmonary function and reduced TAC was independently associated with longitudinal lung function decline [85]. More recently, the same authors reported that TAC is associated with the number of terminal bronchioles as well as with distortion and remodelling of the terminal bronchiole. They suggest that TAC may be a useful imaging biomarker to estimate small airway pathology [86,87]. The histological examination not only confirmed the reduction in terminal bronchioles and the decrease in the luminal areas, but also a reduction in wall volumes and alveolar attachments of terminal, preterminal and pre-preterminal bronchioles with an increased B cell infiltration of these walls [87]. In another paper, ex vivo PRM analysis was compared with in vivo lung tissue measurements of patients with severe COPD treated by lung transplantation and control subjects. There, fSAD identified areas of lung tissue with loss of terminal bronchioli, luminal narrowing and obstruction while emphysema based on PRM correlated with increased airspace size, decreased alveolar surface area and fewer alveolar attachments per terminal bronchiole [88]. Based on this set of publications, PRM can be considered as a useful non-invasive biomarker to detect the earliest pathological changes in COPD.

Independent of early disease detection, the evaluation of regional lung architecture will become extremely important towards a personalised management strategy [89]. Functional respiratory imaging (FRI) allows visualisation and quantification of lung structures and tissues. FRI offers information concerning lobar volumes, airway numbers and volumes, airway resistance and blood vessel volumes at lobe level [90]. 

The recently reported COPD Gene data, aiming to redefine the diagnosis of COPD, are intriguing: they analysed four key disease characteristics—environmental exposure (cigarette smoking), clinical symptoms (dyspnoea and/or chronic bronchitis), chest CT imaging abnormalities (emphysema, gas trapping and/or airway wall thickening) and abnormal spirometry in 8784 current and former smokers participating in the COPD gene. Evidence of COPD progression was based on a change in FEV_1_ beyond the expected normal age-related loss (>350 mL loss over 5 years). Interestingly, abnormal spirometry with or without symptoms was not predictive of spirometric progression, suggesting that spirometry fails to capture and contextualise the extent of disease manifestation. Imaging either in combination with symptoms and/or spirometry was significantly predictive for COPD progression [35].

## 4. Defining COPD in the 21st Century

Health and disease are critical concepts in medicine. While articulating a satisfactory definition of disease is surprisingly difficult, scant attention has been paid to defining disease in clinical medicine. A naturalist conception of disease is that the human body comprises organ systems that have natural functions from which they can depart in many ways: some of these departures are considered harmless, others harmful: determination of bodily malfunction as well as the detrimental effects of the malfunction to human well-being must be objectified by science [91,92]. This view is also reflected in Campbell’s definition of disease as the sum of the abnormal phenomena displayed by a group of living organisms in association with a specified common characteristic or set of characteristics by which they differ from the norm for their species in such a way as to place them at biological disadvantage [93]. Widespread, generalised narrowing of the bronchial airways, persistent or intermittent, was introduced at the CIBA symposium to classify, besides chronic bronchitis, the group of patients with generalised obstructive lung disease [9]. Abnormal functioning of the respiratory system became operationalised and restricted to airflow limitation and the definition of abnormality became based on expert-driven cut-off criteria [14]. The current review illustrates the lack of diagnostic accuracy of this currently applied criterion as well as the insensitivity to detect early abnormalities in the airways and the rest of the respiratory system. A reform of the taxonomy of COPD and COPD-related conditions based on identification of the precise nature of a patient’s problem is urgently needed to move forward towards personalised and precision medicine. On the other hand, lung function trajectories illustrate the important impact of early adulthood lung function leading to the currently applied cut-off of FEV_1_/FVC ratio later in life, resulting in misclassification of these persons as diseased based on spirometry only [39]. Recent CT findings stress the important role of dysanapsis or the mismatch of airway tree calibre to lung size in achieving the FEV_1_/FVC cut-off values [82]. Clinicians must realize that mislabelling someone as diseased will have enormous individual, social and financial implications. In particular, for a disease, labelled for decades as a self-inflicting condition, the label itself will lead to significant individual distress [16]. The concept of pre-COPD for individuals without persistent airflow limitation, but who complain of dyspnoea, cough and/or sputum production in the presence of physiological or radiographic abnormalities, ignores that the presence of airway or alveolar abnormalities is part of the definition of COPD [94,95]. Lumping in of patients with a wide scattering of symptoms and underlying abnormalities without adequate diagnostic labelling will only create more confusion.

Considering that diagnosis is the act of labelling someone as diseased, nosology of chronic respiratory diseases can no longer rely on historical concepts and definitions, but must integrate new scientific and medical discoveries. Imaging as well as new or rediscovered physiology measurements now offer the possibility to obtain an integrated functioning of the respiratory system even in early phases of malfunction. In the future, systems biology coupled with a label-free high-throughput detection could offer new diagnostic tools built on molecular knowledge of the disease. Such a correct identification of respiratory and systemic abnormalities forms the cornerstone for personalised management strategies and will direct better understanding of the complexity of the COPD syndrome. The respiratory community must be convinced that such a trait-based approach will open approaches to early treatment and new research strategies for one of the most disabling diseases. The failure of the simplified, spirometrically based management and research approach justifies a disruptive taxonomic movement to bring this disease into the era of precision medicine.

## References

[1] Collaborators GBDCoD (2018). Global, Regional, and National Age-Sex-Specific Mortality for 282 Causes of Death in 195 Countries and Territories, 1980–2017: A Systematic Analysis for the Global Burden of Disease Study 2017. Lancet.

[2] Lopez A.D., Shibuya K., Rao C., Mathers C.D., Hansell A., Held L.S., Schmid V., Buist S. (2006). Chronic Obstructive Pulmonary Disease: Current Burden and Future Projections. Eur. Respir. J..

[3] Global Strategy for the Diagnosis, Management and Prevention of Chronic Obstructive Pulmonary Disease. https://goldcopd.org/wp-content/uploads/2018/11/GOLD-2019-v1.7-FINAL-14Nov2018-WMS.pdf.

[4] Buist A.S., McBurnie M.A., Vollmer W.M., Gillespie S., Burney P., Mannino D., Menezes A.M., Sullivan S.D., Lee T., Weiss K.B. (2007). International Variation in the Prevalence of COPD (The BOLD Study): A Population-Based Prevalence Study. Lancet.

[5] Murray C.J.L., Vos T., Lozano R., Naghavi M., Flaxman A.D., Michaud C., Ezzati M., Shibuya K., Salomon J.A., Abdalla S. (2012). Disability-Adjusted Life Years (DALYs) for 291 Diseases and Injuries in 21 Regions, 1990–2010: A Systematic Analysis for the Global Burden of Disease Study 2010. Lancet.

[6] Kirsch F., Schramm A., Schwarzkopf L., Lutter J.I., Szentes B., Huber M., Leidl R. (2019). Direct and Indirect Costs of COPD Progression and Its Comorbidities in a Structured Disease Management Program: Results from the LQ-DMP Study. Respir. Res..

[7] Ray S.M., Guarascio A.J., Finch C.K., Self T.H. (2013). The Clinical and Economic Burden of Chronic Obstructive Pulmonary Disease in the USA. Clin. Outcomes Res..

[8] Wacker M., Jörres R., Schulz H., Heinrich J., Karrasch S., Karch A., Koch A., Peters A., Leidl R., Vogelmeier C. (2016). Direct and Indirect Costs of COPD and Its Comorbidities: Results from the German COSYCONET Study. Respir. Med..

[9] (1959). Termininology, Definitions and Classification of Chronic Pulmonary Emphysema and Related Conditions. Thorax.

[10] Snider G.L. (2003). Nosology for Our Day: Its Application to Chronic Obstructive Pulmonary Disease. Am. J. Respir. Crit. Care Med..

[11] Scadding J.G. (1988). Health and Disease: What Can Medicine Do for Philosophy?. J. Med. Ethic..

[12] Seidlein A.-H., Salloch S. (2019). Illness and Disease: An Empirical-Ethical Viewpoint. BMC Med. Ethic..

[13] Twaddle A. (1968). Influence and Illness: Definitions and Definers of Illness Behavior Among Older Males in Providence, Rhode Island. Master’s Thesis.

[14] Pauwels R.A., Buist A.S., Calverley P.M.A., Jenkins C., Hurd S.S. (2001). Global Strategy for the Diagnosis, Management, and Prevention of Chronic Obstructive Pulmonary Disease. Am. J. Respir. Crit. Care Med..

[15] Kerstjens H.A.M. (2004). The GOLD Classification has not Advanced Understanding of COPD. Am. J. Respir. Crit. Care Med..

[16] Temple L.K.F., McLeod R.S., Gallinger S., Wright J.G. (2001). Essays on Science And Society: Defining Disease in the Genomics Era. Science.

[17] Hutchinson J. (1846). On the Capacity of the Lungs, and on the Respiratory Functions, with a View of Establishing a Precise and Easy Method of Detecting Disease by the Spirometer. Med. Chir. Trans..

[18] Tiffenau R., Pinelli A. (1947). Air Curlant et Air Captive dans L’exploration de la Function Ventilatrice Pulmonaire. Paris Med..

[19] Gaensler E.A. (1950). Air Velocity Index: A Numerical Expression of the Functionally Effective Portion of Ventilation. Am. Rev. Tuberc..

[20] Gaensler E.A. (1951). Analysis of the Ventilatory Defect by Timed Capacity Measurements. Am. Rev. Tuberc..

[21] Briscoe W.A., Nash E.S. (1965). The Slow Space in Chronic Obstructive Pulmonary Disease. Ann. N. Y. Acad. Sci..

[22] Bates D. (1989). Respiratory Function in Disease.

[23] Smith T.F., Archie F., Wilson M.D. (1985). Pulmonary Function Testing: Indications and Interpretations.

[24] Pride N., Macklem P., Macklem P., Mead J. (1986). The Respiratory System. Mechanics of Breathing. Handbook of Physiology.

[25] Pellegrino R., Viegi G., Brusasco V., Crapo R.O., Burgos F., Casaburi R., Coates A., Van Der Grinten C.P.M., Gustafsson P., Hankinson J. (2005). Interpretative Strategies for Lung Function Tests. Eur. Respir. J..

[26] Griner P.F., Mayewski R.J., Mushlin A.I., Greenland P. (1981). Selection and Interpretation of Diagnostic Tests and Procedures. Principles and Applications. Ann. Intern. Med..

[27] Hankinson J.L., Odencrantz J.R., Fedan K.B. (1999). Spirometric Reference Values from a Sample of the General U.S. Population. Am. J. Respir. Crit. Care Med..

[28] Mannino D.M., Buist S.A., Vollmer W.M. (2007). Chronic Obstructive Pulmonary Disease in the Older Adult: What Defines Ab-Normal Lung Function?. Thorax.

[29] van Dijk W., Tan W., Li P., Guo B., Li S., Benedetti A., Bourbeau J., CanCOLD Study Group (2015). Clinical Relevance of Fixed Ratio vs Lower Limit of Normal of FEV1/FVC in COPD: Patient-Reported Outcomes from the CanCOLD Cohort. Ann Fam Med..

[30] Bhatt S.P., Balte P.P., Schwartz J.E., Cassano P.A., Couper D., Jacobs D.R., Kalhan R., O’Connor G.T., Yende S., Sanders J.L. (2019). Discriminative Accuracy of FEV1:FVC Thresholds for COPD-Related Hospitalization and Mortality. JAMA.

[31] Celli B.R., Cote C.G., Marin J.M., Casanova C., Montes de Oca M., Mendez R.A., Plata V.P., Cabral H.J. (2004). The Body-Mass Index, Airflow Obstruction, Dyspnea, and Exercise Capacity Index in Chronic Obstructive Pulmonary Disease. N. Engl. J. Med..

[32] Hurst J.R., Anzueto A., Vestbo J. (2017). Susceptibility to Exacerbation in COPD. Lancet Respir Med..

[33] Mohamed Hoesein F.A., Zanen P., Lammers J.W. (2011). Lower Limit of Normal or FEV1/FVC <0.70 in Diagnosing COPD: An EvidenceBased Review. Respir. Med..

[34] Wollmer P., Engström G. (2013). Fixed Ratio or Lower Limit of Normal as Cut-Off Value for FEV1/VC: An Outcome Study. Respir. Med..

[35] Lowe K.E., Regan E.A., Anzueto A., Austin E., Austin J.H., Beaty T.H., Benos P.V., Benway C.J., Bhatt S.P., Bleecker E.R. (2019). COPDGene® 2019: Redefining the Diagnosis of Chronic Obstructive Pulmonary Disease. Chronic Obs. Pulm Dis..

[36] Valenstein P.N. (1990). Evaluating Diagnostic Tests with Imperfect Standards. Am. J. Clin. Pathol..

[37] Tager I.B., Segal M.R., Speizer F.E., Weiss S.T. (1988). The Natural History of Forced Expiratory Volumes: Effect of Cigarette Smoking and Respiratory Symptoms. Am. Rev. Respir. Dis..

[38] Fletcher C., Peto R. (1977). The Natural History of Chronic Airflow Obstruction. BMJ.

[39] Lange P., Celli B.R., Agustí A., Jensen G.B., Divo M., Faner R., Guerra S., Marott J.L., Martinez F.D., Camblor P.M. (2015). Lung-Function Trajectories Leading to Chronic Obstructive Pulmonary Disease. N. Engl. J. Med..

[40] Burrows B., Knudson R.J., Cline M.G., Lebowitz M.D. (1977). Quantitative Relationships between Cigarette Smoking and Ventilatory Function. Am. Rev. Respir. Dis..

[41] Agustí A., Noell G., Brugada J., Faner R. (2017). Lung Function in Early Adulthood and Health in Later Life: A Transgenerational Cohort Analysis. Lancet Respir. Med..

[42] Ross J.C., Castaldi P.J., Cho M.H., Hersh C.P., Rahaghi F.N., Sanchez-Ferrero G.V., Parker M.M., Litonjua A.A., Sparrow D., Dy J.G. (2018). Longitudinal Modeling of Lung Function Trajectories in Smokers with and without Chronic Obstructive Pulmonary Disease. Am. J. Respir. Crit. Care Med..

[43] Doyle L.W., Andersson S., Bush A., Cheong J.L.Y., Clemm H., Evensen K.A.I., Gough A., Halvorsen T., Hovi P., Kajantie E. (2019). Expiratory Airflow in Late Adolescence and Early Adulthood in Individuals Born Very Preterm or with very Low Birthweight Compared with Controls Born at Term or with Normal Birthweight: A Meta-Analysis of Individual Participant Data. Lancet Respir. Med..

[44] Boucherat O., Morissette M.C., Provencher S., Bonnet S., Maltais F. (2016). Bridging Lung Development with Chronic Obstructive Pulmonary Disease. Relevance of Developmental Pathways in Chronic Obstructive Pulmonary Disease Pathogenesis. Am. J. Respir. Crit. Care Med..

[45] Wong P.M., Lees A.N., Louw J., Lee F.Y., French N., Gain K., Murray C.P., Wilson A., Chambers D.C. (2008). Emphysema in Young Adult Survivors of Moderate-to-Severe Bronchopulmonary Dysplasia. Eur. Respir. J..

[46] Tan W.C., Bourbeau J., Hernandez P., Chapman K.R., Cowie R., FitzGerald J.M., Marciniuk D.D., Maltais F., Buist A.S., O’Donnell E.D. (2014). Exacerbation-Like Respiratory Symptoms in Individuals without Chronic Obstructive Pulmonary Disease: Results from a Population-Based Study. Thorax.

[47] Bowler R.P., Kim V., Regan E., Williams A.A., Santorico S.A., Make B.J., Lynch D.A., Hokanson J.E., Washko G.R., Bercz P. (2014). Prediction of Acute Respiratory Disease in Current and Former Smokers with and Without COPD. Chest.

[48] Regan E.A., Lynch D.A., Curran-Everett D., Curtis J., Austin J.H.M., Grenier P.A., Kauczor H.-U., Bailey W.C., DeMeo D.L., Casaburi R.H. (2015). Clinical and Radiologic Disease in Smokers with Normal Spirometry. JAMA Intern. Med..

[49] Woodruff P.G., Barr R.G., Bleecker E., Christenson S.A., Couper D., Curtis J., Gouskova N.A., Hansel N.N., Hoffman E., Kanner R.E. (2016). Clinical Significance of Symptoms in Smokers with Preserved Pulmonary Function. N. Engl. J. Med..

[50] Rodriguez-Roisin R., Han M.K., Vestbo J., Wedzicha J.A., Woodruff P.G., Martinez F.J. (2017). Chronic Respiratory Symptoms with Normal Spirometry. A Reliable Clinical Entity?. Am. J. Respir. Crit. Care Med..

[51] Heard B.E. (1959). Further Observations on the Pathology of Pulmonary Emphysema in Chronic Bronchitics. Thorax.

[52] Morice A.H., Millqvist E., Bieksiene K., Birring S.S., Dicpinigaitis P., Ribas C.D., Boon M.H., Kantar A., Lai K., McGarvey L. (2020). ERS Guidelines on the Diagnosis and Treatment of Chronic Cough in Adults and Children. Eur. Respir. J..

[53] Celli B.R., Wedzicha J.A. (2019). Update on Clinical Aspects of Chronic Obstructive Pulmonary Disease. N. Engl. J. Med..

[54] Levine G., Housley F., MacLeod P., Macklem P.T. (1970). Gas Exchange Abnormalities in Mild Bronchitis and Asymptomatic Asthma. N. Engl. J. Med..

[55] Cotes J.E., Chinn D.J., Quanjer P.H., Roca J., Yernault J.C. (1993). Standardization of the Measurement of Transfer Factor (Diffusing Capacity). Report Working Party Standardization of Lung Function Tests, European Community for Steel and Coal. Official Statement of the European Respiratory Society. Eur. J. Respir. Dis. Suppl..

[56] Welle I., Eide G.E., Bakke P.S., Gulsvik A. (1999). The Single-Breath Transfer Factor for Carbon Monoxide and Respiratory Symptoms in a Norwegian Community Sample. Eur. Respir. J..

[57] Watson A., Joyce H., Hopper L., Pride N.B. (1993). Influence of Smoking Habits on Change in Carbon Monoxide Transfer Factor over 10 Years in Middle Aged Men. Thorax.

[58] Harvey B.-G., Strulovici-Barel Y., Kaner R.J., Sanders A., Vincent T.L., Mezey J.G., Crystal R.G. (2015). Risk of COPD with Obstruction in Active Smokers with Normal Spirometry and Reduced Diffusion Capacity. Eur. Respir. J..

[59] Watson A., Joyce H., Pride N. (2000). Changes in Carbon Monoxide Transfer over 22 Years in Middle-Aged Men. Respir. Med..

[60] Sherrill D.L., Enright P.L., Kaltenborn W.T., Lebowitz M.D. (1999). Predictors of Longitudinal Change in Diffusing Capacity over 8 Years. Am. J. Respir. Crit. Care Med..

[61] Viegi G., Sherrill D.L., Carrozzi L., Di Pede F., Baldacci S., Pistelli F., Enright P. (2001). An 8-Year Follow-up of Carbon Monoxide Diffusing Capacity in a General Population Sample of Northern Italy. Chest.

[62] Storebø M.L., Eagan T.M.L., Eide G.E., Gulsvik A., Thorsen E., Bakke P.S. (2016). Change in Pulmonary Diffusion Capacity in a General Population Sample over 9 Years. Eur. Clin. Respir. J..

[63] Augustin I.M.L., Spruit M.A., Houben-Wilke S., Franssen F.M.E., Vanfleteren L.E.G.W., Gaffron S., Janssen D.J.A., Wouters E.F.M. (2018). The Respiratory Physiome: Clustering Based on a Comprehensive Lung Function Assessment in Patients with COPD. PLoS ONE.

[64] Mead J. (1970). The Lung’s “Quiet Zone”. N. Engl. J. Med..

[65] Rohrer F., Bethe A., Embden G., von Bergmann G. (1925). Physiologie der Atembewegung. Handbuch der Normalen and Pathologischen Physiologie.

[66] Weibel E. (1963). Morphometry of the Human Lung.

[67] Macklem P.T., Mead J. (1967). Resistance of Central and Peripheral Airways Measured by a Retrograde Catheter. J. Appl. Physiol..

[68] Hogg J.C., Macklem P.T., Thurlbeck W.M. (1968). Site and Nature of Airway Obstruction in Chronic Obstructive Lung Disease. N. Engl. J. Med..

[69] Yanai M., Sekizawa K., Ohrui T., Sasaki H., Takishima T. (1992). Site of Airway Obstruction in Pulmonary Disease: Direct Measurement of Intrabronchial Pressure. J. Appl. Physiol..

[70] Hogg J.C., Paré P.D., Hackett T.-L. (2017). The Contribution of Small Airway Obstruction to the Pathogenesis of Chronic Obstructive Pulmonary Disease. Physiol. Rev..

[71] Soares M., Richardson M., Thorpe J., Owers-Bradley J., Siddiqui S. (2019). Comparison of Forced and Impulse Oscillometry Meas-urements: A Clinical Population and Printed Airway Model Study. Sci. Rep..

[72] Cooxson H.O., Leipsic J., Parraga G., Sin D.D. (2014). Using Pulmonary Imaging to Move Chronic Obstructive Pulmonary Disease Beyond FEV1. Am. J. Respir. Crit. Care Med..

[73] Labaki W., Martinez C.H., Martinez F.J., Galbán C.J., Ross B.D., Washko G.R., Barr R.G., Regan E., Coxson H.O., Hoffman E. (2017). The Role of Chest Computed Tomography in the Evaluation and Management of the Patient with Chronic Obstructive Pulmonary Disease. Am. J. Respir. Crit. Care Med..

[74] Remy-Jardin M., Remy J., Boulenguez C., Sobaszek A., Edme J.L., Furon D. (1993). Morphologic Effects of Cigarette Smoking on Airways and Pulmonary Parenchyma in Healthy Adult Volunteers: CT Evaluation and Correlation with Pulmonary Function Tests. Radiology.

[75] Mohamed Hoesein F.A., Zanen P., de Jong P.A., Firdaus A.A., van Ginneken B., Boezen H.M., Groen H.J.M., Oudkerk M., de Koning H.J., Postma D.S. (2013). Rate of Progression of CT-Quantified Emphysema in Male Current and Ex-Smokers: A Follow-Up Study. Respir. Res..

[76] Regan E.A., Hokanson J.E., Murphy J.R., Make B., Lynch D.A., Beaty T.H., Curran-Everett D., Silverman E.K., Crapo J.D. (2010). Genetic Epidemiology of COPD (COPDGene) Study Design. COPD: J. Chronic Obstr. Pulm. Dis..

[77] Tan W.C., Hague C.J., Leipsic J., Bourbeau J., Zheng L., Li P.Z., Sin D.D., Coxson H.O., Kirby M., Hogg J.C. (2016). Findings on Thoracic Computed Tomography Scans and Respiratory Outcomes in Persons with and without Chronic Obstructive Pulmonary Disease: A Population-Based Cohort Study. PLoS ONE.

[78] Galbán C.J., Han M.K., Boes J.L., Chughtai K.A., Meyer C.R., Johnson T.D., Galbán S., Rehemtulla A., Kazerooni E.A., Martinez F.J. (2012). Computed Tomography–Based Biomarker Provides Unique Signature for Diagnosis of COPD Phenotypes and Disease Progression. Nat. Med..

[79] Bhatt S.P., Soler X., Wang X., Murray S., Anzueto A.R., Beaty T.H., Boriek A.M., Casaburi R., Criner G.J., Diaz A.A. (2016). Association between Functional Small Airway Disease and FEV1Decline in Chronic Obstructive Pulmonary Disease. Am. J. Respir. Crit. Care Med..

[80] Criner R.N., Hatt C.R., Galbán C.J., Kazerooni E.A., Lynch D.A., McCormack M.C., Casaburi R., MacIntyre N.R., Make B.J., Martinez F.J. (2019). Relationship between Diffusion Capacity and Small Airway Abnormality in COPDGene. Respir. Res..

[81] Young A.L., Bragman F.J.S., Rangelov B., Han M.K., Galbán C.J., Lynch D.A., Hawkes D.J., Alexander D.C., Hurst J.R., Crapo J.D. (2020). Disease Progression Modeling in Chronic Obstructive Pulmonary Disease. Am. J. Respir. Crit. Care Med..

[82] Smith B.M., Kirby M., Hoffman E., Kronmal R.A., Aaron S.D., Allen N., Bertoni A., Coxson H.O., Cooper C., Couper D.J. (2020). Association of Dysanapsis with Chronic Obstructive Pulmonary Disease Among Older Adults. JAMA.

[83] McDonough J., Yuan R., Suzuki M., Seyednejad N., Elliott W.M., Sanchez P.G., Wright A.C., Gefter W.B., Litzky L., Coxson H.O. (2011). Small-Airway Obstruction and Emphysema in Chronic Obstructive Pulmonary Disease. N. Engl. J. Med..

[84] Koo H.-K., Vasilescu D.M., Booth S., Hsieh A., Katsamenis O., Fishbane N., Elliott W.M., Kirby M., Lackie P., Sinclair I. (2018). Small Airways Disease in Mild and Moderate Chronic Obstructive Pulmonary Disease: A Cross-Sectional Study. Lancet Respir. Med..

[85] Kirby M., Tanabe N., Tan W.C., Zhou G., Obeidat M., Hague C.J., Leipsic J., Bourbeau J., Sin D.D., Hogg J.C. (2018). Total Airway Count on Computed Tomography and the Risk of Chronic Obstructive Pulmonary Disease Progression. Findings from a Population-based Study. Am. J. Respir. Crit. Care Med..

[86] Kirby M., Tanabe N., Vasilescu D.M., Cooper J.D., McDonough J.E., Verleden S.E., Vanaudenaerde B.M., Sin D.D., Tan W.C., Coxson H.O. (2020). Computed Tomography Total Airway Count is Associated with Number of Mi-cro-CT Terminal Bronchioles. Am. J. Respir. Crit. Care Med..

[87] Tanabe N., Vasilescu D.M., Kirby M., Coxson H.O., Verleden S.E., Vanaudenaerde B.M., Kinose D., Nakano Y., Paré P.D., Hogg J.C. (2018). Analysis of Airway Pathology in COPD Using a Combination of Computed Tomography, Micro-Computed Tomography and Histology. Eur. Respir. J..

[88] Vasilescu D.M., Martinez F.J., Marchetti N., Galbán C.J., Hatt C., Meldrum C.A., Dass C., Tanabe N., Reddy R.M., Lagstein A. (2019). Noninvasive Imaging Biomarker Identifies Small Airway Damage in Severe Chronic Obstructive Pulmonary Disease. Am. J. Respir. Crit. Care Med..

[89] Burrowes K.S., Doel T., Brightling C. (2014). Computational Modeling of the Obstructive Lung Diseases Asthma and COPD. J. Transl. Med..

[90] De Backer J.W., Vos W.G., Vinchurkar S.C., Claes R., Drollmann A., Wulfrank D., Parizel P.M., Germonpré P., De Backer W. (2010). Validation of Computational Fluid Dynamics in CT-Based Airway Models with SPECT/CT. Radiology.

[91] Boorse C. (1975). On the Distiction Between Disease and Illness. Philosophy Public Affairs.

[92] Boorse C. (1977). Health as a Theoretical Concept. Philos. Sci..

[93] Campbell E.J., Scadding J.G., Roberts R.S. (1979). The Concept of Disease. Br. Med. J..

[94] Agustí A., Celli B. (2017). Natural History of COPD: Gaps and Opportunities. ERJ Open Res..

[95] Han M.K., Agusti A., Celli B.R., Criner G.J., Halpin D.M.G., Roche N., Papi A., Stockley R.A., Wedzicha J., Vogelmeier C.F. (2021). From GOLD 0 to Pre-COPD. Am. J. Respir. Crit. Care Med..

